# Commentary: Effects of combined treatment with transcranial and peripheral electromagnetic stimulation on performance and pain recovery from delayed onset muscle soreness induced by eccentric exercise in young athletes. A randomized clinical trial

**DOI:** 10.3389/fphys.2024.1380261

**Published:** 2024-05-10

**Authors:** Balázs Sonkodi

**Affiliations:** ^1^ Department of Health Sciences and Sport Medicine, Hungarian University of Sports Science, Budapest, Hungary; ^2^ Department of Sports Medicine, Semmelweis University, Budapest, Hungary

**Keywords:** delayed-onset muscle soreness, PIEZO2 channel, ASIC3 channels, ASIC2, VGLUT1/2, electromagnetic stimulation

## Introduction

The finding that combined treatment with transcranial and peripheral electromagnetic stimulation (TES and PES, respectively) improves performance and pain recovery from delayed-onset muscle soreness (DOMS) is a significant step forward not only in the treatment of DOMS (Keriven et al., 2023a) but also in the understanding of the mechanism of this mysterious pain condition. Keriven et al. proposed that this paired-associative treatment method is alleviating the compression on proprioceptive afferent terminals based on the recent acute compression proprioceptive axonopathy theory of DOMS by Sonkodi et al. (2020), Sonkodi et al. (2021b), and Sonkodi (2022). However, this neurocentric DOMS theory was inadequately cited in Keriven et al. (2023a) since Stifani (2014), Radovanovic et al. (2015), and Colon et al. (2017) did not formulate any theories on the DOMS mechanism, as indicated in the referred paper.

Keriven et al. selected intriguingly well the treatment methods and the goal of the study (Keriven et al., 2023a), especially based on the earlier results of Milanovic et al. (2011), Matsuo et al. (2022) and Qin et al. (2023). Yet, the question rightly arises why only the combined TES and PES treatment is effective in DOMS, in contrast to the unsuccessful PES-only treatment method (Keriven et al., 2023b). Correspondingly, Khataei and Benson adequately addressed that the understanding of the molecular mechanism of the barriers to exercise, more specifically to DOMS, is at high need (Khataei and Benson, 2023), including the elucidation of the aforementioned question.

## ASIC3, Piezo2, ASIC2, and VGLUT in the DOMS molecular mechanism

Khataei and Benson demonstrated that acid-sensing ion channel 3 (ASIC3) plays a protective role in DOMS (Khataei and Benson, 2023). Indeed, ASIC3 is present on Type II proprioceptive afferents in the dorsal root ganglion and extrafusal space. Moreover, the ASIC3 ion channel is shown to participate in proprioceptive mechanotransduction (Lin et al., 2016), in addition to Piezo2 being the principal ion channel (Woo et al., 2015).

It is important to note that DOMS is theorized to be a bi-phasic and bi-compartmental microinjury mechanism, meaning the involvement of both the intra- and the extrafusal space, according to the referred acute compression proprioceptive axonopathy theory of DOMS (Sonkodi et al., 2020; Sonkodi, 2022). In agreement with this theory, the primary damage is suggested to be an acute Piezo2 channelopathy in the muscle spindle, lasting 1–3 days (Sonkodi et al., 2021b; Sonkodi, 2022). It is worth noting that this primary damage of DOMS is proposed to be associated with the impairment of glutamate vesicular release machinery at the intrafusal proprioceptive terminal (Sonkodi et al., 2021b; Sonkodi, 2022) as well, and they may initiate the loss of the static phase-of-firing encoding on Type Ia fibers concomitantly (Sonkodi et al., 2022).

One consequence, among others, of this intrafusal transient proprioceptive terminal Piezo2 channelopathy is suggested to be a proprioceptive switch, meaning the lost static phase-of-firing encoding of the intrafusal Type Ia fibers are conveyed on by only Type II proprioceptive fibers in a compensatory fashion (Sonkodi, 2021). As a result of this primary damage of DOMS, the theoretical switch of static phase-of-firing encoding from monosynaptic large Type Ia fiber signaling to a polysynaptic lower Type II proprioceptive fiber is demonstrated in the significant increase in the medium latency response of the stretch reflex (Sonkodi et al., 2022). This switch is suggested to be a preprogrammed secondary compensatory pathway for stabilizing postural control due to the primary damage of DOMS (Sonkodi et al., 2021b; Sonkodi, 2021). The transiently lost static phase-of-firing encoding on Type Ia fibers is also proposed to result in the transient loss of vesicular glutamate transporter 1(VGLUT1)/Ia synapses on motoneurons (Sonkodi et al., 2022), based on observations from nerve injury studies (Alvarez et al., 2011; Bullinger et al., 2011).

An even more recent novel theory postulates that proprioceptive terminal Piezo2-initiated proton-based frequency coupling through VGLUT2 may provide a long suspected ultrafast long-range signaling for the synchronization of the low-frequency glutamatergic cell surface membrane oscillations in order to provide proprioceptive input to hippocampal theta rhythm encoding (Sonkodi, 2023a; 2024). This theory is analogous to the earlier coupled oscillator model that suggested the entrainment of the imposed forcing intrafusal Type Ia afferent peripheral oscillator to the oscillator(s) of the central nervous system (CNS) (Cathers et al., 2006). Hence, as a result of the primary damage of DOMS, not only VGLUT1 may disconnect transiently on motoneurons, as was suggested by Sonkodi et al. (2022), but also VGLUT2 disconnection could be another consequence in the CNS, leading to impaired proprioception (Sonkodi, 2023a; Sonkodi, 2024). Highly indicative of this VGLUT1/2 disconnection theory is the finding that motor output is misjudged and disturbed when the “exercised arm acted as its own reference” after eccentric and isometric exercise (Philippou et al., 2010). Even Philippou et al. interpreted this lack of congruence as the impaired motor control of damaged muscle is mismatched with the central motor command (Philippou et al., 2010).

It should be noted that ASIC2 ion channels are also present on proprioceptive Type Ia terminals (Simon et al., 2010), and most likely, they provide the proton-based signaling pathway between Piezo2 and VGLUT (Sonkodi, 2024). However, the low-frequency Schottky barrier semiconductor diode feature of Piezo2 may be the one providing the control of fine movements (Sonkodi, 2023a; Sonkodi, 2024) in addition to proprioceptive signal generation, but an intimate co-functioning of Piezo2 and ASIC2 is suspected in this process (Bornstein et al., 2023; Sonkodi, 2024). Not surprisingly, lost ASIC2 function alters muscle spindle-derived stretch responses and motor coordination (Bornstein et al., 2023). Hence, probably not all ASICs could be excluded as DOMS requirement, like proposed by Khataei and Benson, but excluding ASIC3 is certainly a major step forward in the molecular understanding of DOMS (Khataei and Benson, 2023).

## Discussion

The aforementioned simultaneous transient central synaptic disconnection of proprioceptors from motoneurons through the loss of VGLUT1/Ia synapses and VGLUT2 disconnection in the CNS due to the DOMS effect (Sonkodi, 2023a; Sonkodi, 2024) could be the missing link why only the combined TES and PES treatment is effective. Accordingly, the electromagnetic stimulation may compensate for the lost essential proton-signaled proprioceptive feedback loops on upper and lower motoneurons but only if the stimulation is conveyed on both motoneuron territories. Consequently, this muscle spindle-derived Piezo2-generated proprioceptive input appears to be essential for motoneurons. Otherwise, in the absence of this signaling, not only the medium latency response of the stretch reflex will be delayed (Sonkodi et al., 2022) but also the M-wave latency will be increased transiently as well on motoneurons in DOMS (Kouzaki et al., 2016).

In support of the aforementioned essentiality, the irreversibly and progressively lost function of Piezo2 in proprioceptive terminals is suggested to be one critical underlying process in the amyotrophic lateral sclerosis (ALS) pathomechanism (Sonkodi, 2021; Sonkodi and Hortobagyi, 2022; Sonkodi, 2023b; Sonkodi, 2024). Moreover, animal studies also show that Piezo2-knockout mice do not survive after delivery; hence, the homeostatic gatekeeper function of Piezo2 on somatosensory neurons is essential in order to protect the CNS (Ranade et al., 2014; Volkers et al., 2015).

Correspondingly, any systemic direct or indirect microdamage that leads to the irreversible detachment or the impediment of the regeneration of Piezo2-containing proprioceptive terminals seems to be incompatible with life sustainment (Sonkodi and Hortobagyi, 2022). The paired-associative combined TES and PES treatment is suggested to compensate for the transiently lost essential Piezo2-generated proton-based ultrafast proprioceptive sensory feedback on motoneurons in DOMS and possibly in the early symptomatic stage of ALS as well (Sonkodi, 2024). Furthermore, it is no surprise that ASIC3-null mice suffer more muscle damage due to DOMS-inducing exercise (Khataei and Benson, 2023) because not only the sensing of longitudinal secondary hyperalgesia could be lost (Ikeuchi et al., 2008; Niibori et al., 2020; Sonkodi et al., 2021c) but also the secondary compensatory proprioceptive protection is also lost, and ASIC3 is meant to buffer the primary damage of primary proprioceptor or the proprioceptive Type Ia terminal Piezo2 channelopathy in DOMS.

In summary, the effectiveness of this paired-associative TES and PES treatment in DOMS substantiates the importance of the underlying neural involvement of this perplexing, but unknown, mechanism. However, additional studies are needed as Keriven et al. (2023a) rightly indicated. The issue of other neural non-contact injuries (Sonkodi et al., 2021a), aging, and the longitudinal effect of this proposed combined TES and PES treatment method should be investigated in the future as well.
